# Oxidized carbon black nanoparticles induce endothelial damage through C-X-C chemokine receptor 3-mediated pathway

**DOI:** 10.1016/j.redox.2021.102161

**Published:** 2021-10-04

**Authors:** Nairrita Majumder, Murugesan Velayutham, Dimitrios Bitounis, Vamsi K. Kodali, Md Habibul Hasan Mazumder, Jessica Amedro, Valery V. Khramtsov, Aaron Erdely, Timothy Nurkiewicz, Philip Demokritou, Eric E. Kelley, Salik Hussain

**Affiliations:** a Department of Physiology and Pharmacology, West Virginia University, School of Medicine, USA; b Center for Inhalation Toxicology (iTOX), West Virginia University, School of Medicine, USA; c Department of Biochemistry, West Virginia University, School of Medicine, USA; d Center for Nanotechnology and Nanotoxicology, Department of Environmental Health, T.H. Chan School of Public Health, Harvard University, Boston, MA, USA; e National Institute for Occupational Safety and Health, Morgantown, WV, USA

**Keywords:** Nanoparticle carbon black, Ozone, Oxidized carbon black, Electron paramagnetic spectroscopy, Macrophage, Endothelial cells

## Abstract

Oxidation of engineered nanomaterials during application in various industrial sectors can alter their toxicity. Oxidized nanomaterials also have widespread industrial and biomedical applications. In this study, we evaluated the cardiopulmonary hazard posed by these nanomaterials using oxidized carbon black (CB) nanoparticles (CB_ox_) as a model particle.

Particle surface chemistry was characterized by X-ray photo electron spectroscopy (XPS) and Fourier-transform infrared spectroscopy (FTIR). Colloidal characterization and *in vitro* dosimetry modeling (particle kinetics, fate and transport modeling) were performed. Lung inflammation was assessed following oropharyngeal aspiration of CB or oxidized CB_ox_ particles (20 μg per mouse) in C57BL/6J mice. Toxicity and functional assays were also performed on murine macrophage (RAW 264.7) and endothelial cell lines (C166) with and without pharmacological inhibitors. Oxidant generation was assessed by electron paramagnetic resonance spectroscopy (EPR) and via flow cytometry. Endothelial toxicity was evaluated by quantifying pro-inflammatory mRNA expression, monolayer permeability, and wound closure.

XPS and FTIR spectra indicated surface modifications, the appearance of new functionalities, and greater oxidative potential (both acellular and *in vitro*) of CB_ox_ particles. Treatment with CB_ox_ demonstrated greater *in vivo* inflammatory potentials (lavage neutrophil counts, secreted cytokine, and lung tissue mRNA expression) and air-blood barrier disruption (lavage proteins). Oxidant-dependent pro-inflammatory signaling in macrophages led to the production of CXCR3 ligands (CXCL9,10,11). Conditioned medium from CB_ox_-treated macrophages induced significant elevation in endothelial cell pro-inflammatory mRNA expression, enhanced monolayer permeability and impairment of scratch healing in CXCR3 dependent manner.

In summary, this study mechanistically demonstrated an increased biological potency of CB_ox_ particles and established the role of macrophage-released chemical mediators in endothelial damage.

## Highlights

• Exposure to ozone oxidizes carbon black nanoparticles.
• Oxidized carbon black particles demonstrate enhanced oxidant production.
• Oxidized carbon black particles exposed macrophages secreted CXCL9/10/11 activate CXCR3 on endothelial cells.
• CXCR3 activation mediate increased endothelial monolayer permeability and migration impairment.

## Graphical abstract

**Figure:**
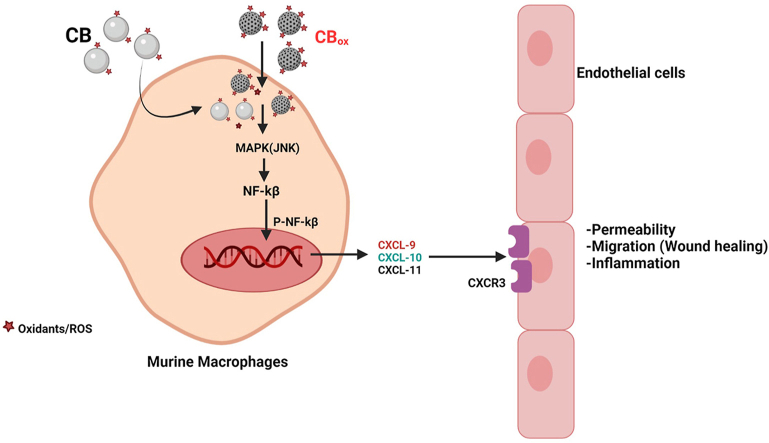

## 1 Introduction

Oxidation of nanomaterials such as carbon black (CB) alters their functional characteristics and creates opportunities for biomedical and industrial applications [1]. Oxidized CB particles can deliver macromolecules to cells (*e.g.,* virus neutralization monoclonal antibodies), enhance nano/microparticle uptake and are postulated as an effective antigen delivery system for targeting cell-mediated immune response [[2], [3], [4], [5]]. Ozone (O_3_)-treated carbon (CB_ox_) is an attractive material for the construction of electrochemical capacitors [6] while oxidation of CB nanoparticles (NP) has shown potential for the generation of graphene quantum dots [7,8]. Moreover, treatment of carbon-based interfacial materials (polymer support interface) with O_3_ with or without ultraviolet (UV) light/heat is considered an efficient way to improve the interface compatibility and mechanical properties [9]. These diverse applications reflect an increased potential for human exposure. Therefore, oxidized CB particles are a material of interest to evaluate potential health issues that may arise from their exposure.

Significant occupational and environmental exposure potential exist for engineered CB. CB NP are among the most widely produced nanomaterials with a variety of applications in pigments, paints, inks/toner and reinforcing agents in polymers [[10], [11], [12], [13]]. Current worldwide production of CB is estimated to be 8.1 million metric tons per year with a market value of $13 billion for applications (carbon black user's guide) [14]. CB is classified as a possible human carcinogen (2B) by the International Agency for Research on Cancer (IARC) [15]. In addition to occupational inhalation exposures (from mining and production), the use of CB in toner and as a rubber reinforcing material creates environmental inhalation exposure scenarios [16]. Occupational inhalation exposure to CB is associated with lung and vascular complications [[17], [18], [19], [20]]. In addition to pulmonary toxicity, significant genotoxicity and reproductive/developmental toxicity are also reported through direct and indirect mechanisms [[21], [22], [23]]. Moreover, CB is widely used as a model for environmental ultrafine particulate exposures [24].

CB_ox_ (sometimes referred to as aged black carbon or ozone-oxidized black carbon) has been shown to induce lung inflammation through PI3K/AKT, MAPK4 and IL-33 pathways as well as result in changes in immune cell proportions in murine lymph nodes [[25], [26], [27], [28]]. Moreover, by utilizing *in vitro* systems [human lung bronchial and alveolar type-2-like cell lines (16HBE140, A549)] it was demonstrated that O_3_-exposed particles induce greater transcriptomic dysregulation, DNA damage, and mitochondrial dysfunction [[29], [30], [31]]. Another study utilizing a murine macrophage cell line (MH-S) failed to reproduce the increased inflammatory potential of CB_ox_ particles [10]. Despite numerous studies, the impact of these particles on endothelial cells and role of macrophage-derived factors in these responses is still not known.

Given the robust association of CB and O_3_ with adverse cardiovascular outcomes and increased inflammatory potential of O_3_-interacted CB, it is plausible that phagocyte-released, soluble inflammatory mediators may induce *in vitro* endothelial changes. While there are large number of studies that report *in vitro* and in vivo toxicity of CB nanoparticles [[32], [33], [34], [35], [36], [37]], there are only a handful that investigate the mechanisms of endothelial dysfunction [[38], [39], [40]]. However, mechanistic studies explaining the impact of CB_ox_ NPs on the endothelial cell function are lacking. Therefore, we evaluated if CB_ox_ particles cause *in vitro* endothelial dysfunction (increased monolayer permeability and impaired migration) and deciphered the mechanism of toxicity by showing that this occurs via inflammatory mediators released by macrophages.

## 2 Materials and methods

### 2.1 CB nanoparticles and CBox particle generation

CB NPs (Printex 90®, a gift from Evonik, Frankfurt, Germany) were used as base material. CB_ox_ particles were generated using an animal inhalation exposure system which was originally developed by the authors to perform co-exposures [41]. In this system, CB particles were aerosolized using a modified high-pressure acoustical generator (HPAG, IEStechno, Morgantown, WV). O_3_ was produced by passing pure oxygen through a corona discharge type O_3_ generator (HTU500AC, Ozone Solutions, Hull, IA). To collect the particles, CB aerosol (10 mg/m^3^) was mixed with ozone (2 ppm) for 3 h and then the suspended particles were collected on polycarbonate membrane filters. The particles were removed from filters by agitation without any chemical extraction. Elemental composition of particle surfaces was analyzed by X-Ray Photoelectron Spectroscopy (XPS) (Physical Electronics PHI 5000 VersaProbe XPS/UPS). Fourier-transform infrared spectroscopy (FTIR) was performed using a Digilabs FTS 7000 FTIR system. Elemental composition of particle surfaces was analyzed by X-Ray Photoelectron Spectroscopy (XPS) (Physical Electronics PHI 5000 VersaProbe XPS/UPS). A detailed deconvolution of O1s and C1 peaks from XPS data was performed using Peakfit v4.12 (Systat Software Inc. SannJose CA).

### 2.2 Dispersion preparation, colloidal characterization, and dosimetry analyses

The dispersion preparation, colloidal characterization, and dosimetric analyses of CB and CB_ox_ particle samples was performed according to protocols described in detail elsewhere [[42], [43], [44], [45], [46], [47], [48]]. In summary, 1.0 mL of CB or CB_ox_ suspensions at 0.5 mg/mL in deionized (DI) water underwent sonication (Branson Sonifier S-450D, 400 W, with Branson 3-in. cup-horn, power delivered: 1.26 W) and 30 s high-speed vortexing. Following each round of cup-horn sonication & vortexing, the hydrodynamic diameter (z-average) of CB or CB_ox_ particles was measured by dynamic light scattering (DLS, Zetasizer Nano ZS, Malvern UK). This step was repeated until dH changed by less than 5% at which point the delivered acoustic energy is termed critical delivered sonication energy (DSEcr).

Once sonicated at their respective DSE_cr_, CB and CB_ox_ were added to fully supplemented Dulbecco Modified Eagles Medium (DMEM) (10% vol fetal bovine serum (FBS) and 100 units of Pen-Strep) at final concentration of 0.1 mg/mL. The hydrodynamic diameter (dH), polydispersity index, zeta potential, and conductivity of the particle suspensions in cell growth medium and DI water were measured for both CB and CB_ox_.

The effective densities of the CB and CB_ox_ particle samples in fully supplemented DMEM were experimentally measured according to the volumetric centrifugation method previously described by the authors [44]. The particokinetics of CB and CBox were calculated using the distorted grid (DG) model executed on MATLAB (MathWorks, Massachusetts, USA) to calculate the fraction of administered CB or CBox particle mass (f_D_) delivered to the surface of cells as a function of exposure time and well geometry, according to a method previously implemented by the authors [42,49].

### 2.3 Electron Paramagnetic Resonance (EPR) spectroscopy

EPR spin probe 1-hydroxy-3-carboxymethyl-2,2,5,5-tetramethyl-pyrrolidine (CMH) was purchased from Enzo Life Sciences. EPR spectra were recorded using a Bruker EMXnano spectrometer (Bruker BioSciences, Billerica, MA, USA) operating at X-band with a 100 kHz modulation frequency as described previously [41,50].

*Acellular Reactive Oxidant Generation on Particle Surface*: EPR spectroscopy was used to measure the oxidizing potential of CB and CB_ox_ particles using spin probe CMH. Briefly, particles (50 μg/mL) were incubated with EPR spin probe 1-hydroxy-3-carboxymethyl-2,2,5,5 tetramethyl-pyrrolidine (0.2 mM, CMH, EPR silent) for 30 min at 37 °C. CMH is oxidized by reactive species on the surface of the CB particles to 3-carboxymethyl-2,2,5,5- tetramethyl-pyrrolidinyloxy radical (CM•; EPR active). After incubation, samples were immediately frozen in liquid nitrogen and stored at −80 °C until the EPR experiments were carried out.

To study the oxidizing potential of CB and CB_ox_ particles on biological antioxidants such as ascorbate, the particles were incubated with sodium ascorbate (1 mM) in phosphate buffer (10 mM, pH 7.4) for 30 min at 37 °C. After incubation, samples were immediately frozen in liquid nitrogen and stored at −80 °C until the EPR experiments were carried out. Ascorbate is oxidized by reactive species on the surface of the CB particles to ascorbate radical. EPR spectroscopy was used to measure the formation of ascorbate radical [51].

*Reactive Oxidants Generation by Particles* in Serum: The formation of reactive oxidant species in the serum was also measured using CMH, as described before [50]. Briefly, samples were exposed to vehicle, CB and CB_ox_ in human serum (Sigma-Aldrich, St. Louis, MO; Cat #P2918) at the concentration of 50 μg/mL for 5 min at 37 °C, followed by incubation with EPR spin probe CMH (0.2 mM) for 30 min at 37 °C. After incubation, samples were immediately frozen in liquid nitrogen and stored at −80 °C until EPR experiments were carried out.

*Reactive Oxidants Generation by Particles in Macrophages:* RAW 264.7 macrophages were treated with vehicle, CB and CB_ox_ at a concentration of 50 μg/mL for 4 h, followed by washing with Chelex (Sigma-Aldrich, Cat #C7901)-treated PBS (pH 7.4) and incubation with CMH (0.2 mM) in Chelex-treated PBS for 30 min at 37 °C. After incubation, the cells were separated from dishes using a cell lifter and cells plus PBS were snap frozen in liquid nitrogen and stored at −80 °C for further EPR experiments.

At the time of EPR measurements, liquid samples were thawed and loaded (50 μL) into glass capillary tubes (Ref: 9600150; Hirschmann Laborgerate GmbH & Co. KG, D-74246 Eberstadt, Germany) that were sealed on one end using Critoseal clay and placed inside the 4 mm (O.D.) EPR quartz tube (Cat log: 707-SQ-250 M; Wilmad LabGlass, Vineland, NJ, USA). The quartz tube was positioned inside the resonator/cavity and EPR spectra were recorded at room temperature. The following EPR instrument settings were used: microwave frequency, 9.615 GHz; center field, 3425 G; sweep width, 100 G; microwave power, 20 mW; modulation amplitude, 0.5 G; modulation frequency, 100 kHz; receiver gain, 60 dB; time constant, 5 ms; conversion time, 15 ms, sweep time, 30 s; number of scans, 1. Data acquisition was performed using Bruker Xenon_nano software. The signal intensity was generated using first peak (low field) height of the EPR spectrum. Data processing was performed using GraphPad Prism 8 (GraphPad software, San Diego, CA).

### 2.4 Ferric Reducing Ability of Serum (FRAS) assay

Complimentary to EPR approach, the reactivity/acellular oxidative potential of the particulates was also evaluated using Ferric Reducing Ability of Serum (FRAS). FRAS measures the oxidant damage/antioxidant depletion in human serum, a rich source of antioxidants that can help evaluate the oxidation induced by multiple chemically distinct oxidants. This approach has been previously used to evaluate the oxidative potential of various engineered nanomaterial [52,53]. Human serum was rapidly thawed and exposed to particulate at 5 mg/mL. The particulate containing solution was briefly sonicated and incubated at 37 °C for 3 h on an orbital shaker set at 450 RPM. The particulate was separated from the serum by centrifuging the mixture at 14,000 g for 30 min. To determine the oxidation, 50 μl of the serum supernatant was reacted with 1 mL of the FRAS solution. The FRAS solution which is a mixture of 10:1:1 vol/vol mixture of A: B: C reagent solutions was mixed right before reacting it with the serum. Reagent “A” consists of 14 mM sodium acetic trihydrate and 176 μM glacial acetic acid (Alfa Aesar, Haverhill, MA; Cat # 36289) in deionized water. Reagent “B” consists of 10.1 mM TPTZ (2,4,6-tri(2-pyridyl)-s-triazine) Sigma-Aldrich, Cat #T1253) and 1 mM HCL in deionized water. Reagent “C” consist of 20 mM FeCl_3_·6H_2_O (Sigma-Aldrich, Cat # 44944) in deionized water. The change in color was quantified by reading the absorption at 586 nm and change in antioxidants was plotted as % change from control serum.

### 2.5 Murine model

C57BL/6J male mice (8 weeks old) were purchased from Jackson Laboratory (Bar Harbor, ME) and acclimated at the West Virginia University (WVU) Animal Care Facility for a week before exposure. All animals were maintained in a room with a 12-h light/dark cycle and provided chow and water ad libitum. All animal procedures were approved by the WVU Institutional Animal Care and Use Committee) that is an AAALAC accredited program. CB (CB collected after aerosolization without reaction with ozone) and CB_ox_ particles were dispersed in freshly collected lavage fluid (from naïve mice) and administered (20 μg in 50 μl volume) by oropharyngeal aspiration. We exposed 5 animals in each treatment group. Euthanasia was performed via intraperitoneal injection of Fatal Plus (250 mg/kg) at 24 h post exposure.

### 2.6 RAW 264.7 and RAW-Blue™ cell culture

RAW 264.7 murine macrophages (ATCC® TIB-71™) and RAW-Blue™ Cells (InvivoGen, San Diego, CA) were cultured in Dulbecco's Modified Eagle's Medium (DMEM) (Sigma-Aldrich St. Louis, MO), supplemented with 10% fetal bovine serum (R&D Systems, Minneapolis, MN), 1% Penicillin-Streptomycin (Gibco™, Carlsbad, CA) 1% antimycotic (Gibco™, Carlsbad, CA), in 5% CO_2_ and 37 °C. RAW-Blue™ cells are derived from RAW 264.7 macrophages with chromosomal integration of a secreted embryonic alkaline phosphatase (SEAP) reporter construct inducible by NF-κB and AP-1. Cells were treated with CB particles (50 μg/mL) and CB_ox_ particles (50 μg/mL) in DMEM (without phenol red) (Sigma-Aldrich St. Louis, MO).

### 2.7 Murine endothelial cell culture

Yolk sac-endothelial cells- C166 (ATCC® CRL-2581™) were cultured in DMEM cell culture medium (Sigma-Aldrich St. Louis, MO), supplemented with 10% fetal bovine serum (R&D Systems, Minneapolis, MN), 1% Penicillin-Streptomycin (Gibco™, Carlsbad, CA), 1% antimycotic (Gibco™, Carlsbad, CA), in 5% CO_2_ and 37 °C. Cells were exposed using a well-established conditioned medium approach. Briefly, media was collected from vehicle, CB, CB_ox_ exposed RAW 264.7 cells after 24 h exposure. A 1:4 dilution of conditioned medium to fresh medium was then used to expose endothelial cells.

### 2.8 Bronchoalveolar Lavage Fluid (BALF) collection and analyses

Following euthanasia, approximately 1 mL ice cold sterile PBS was instilled through a tracheal canula into the lungs three times. The cells were counted in the lavage fluid and pelleted by centrifugation at 600 RPM for 5 min at 4 °C and used for cytospin preparation using Cytospin® (Thermo Fisher Scientific, Waltham, MA) for differential counts. Cells were stained in Hema 3 (Fisher Scientific, Pittsburgh, PA). The lavage fluid supernatant was stored at −80 °C for later investigation. Lavage protein content were quantified using Pierce BCA kit (Thermo Fisher Scientific, Waltham, MA) according to manufacturer's recommendations.

### 2.9 Enzyme Linked Immunosorbent Assay (ELISA)

ELISA assays were performed to quantify keratinocyte chemoattractant (KC), tumor necrosis factor-α (TNF-α), interleukin-6 (IL-6), interleukin-13 (IL-13), interleukin-1ꞵ (IL-1β), thymic stromal lymphopoietin (TSLP) and chemokine ligand 9 (CXCL9) using Duoset sandwich ELISA assay kits (R&D Systems, Minneapolis, MN) according to manufacturer's recommendations. The limit of detection for these assays are as follows: IL-1β (1000 pg/mL – 15.6 pg/mL), TNF-α (2000 pg/mL – 31.3 pg/mL), KC (100 pg/mL – 15.6 pg/mL), IL-6 (1000 pg/mL – 15.6 pg/mL), IL-13 (4000 pg/mL – 62.5 pg/mL), TSLP (1000 pg/mL – 15.6 pg/mL), CXCL9 (1000 pg/mL – 15.6 pg/mL). The quantification was done as previously described [54]. Briefly, standard curve for each cytokine was generated using known serially diluted protein concentrations. This standard curve and unknown samples were processed using ELISA methodology as provided by the manufacturer. The unknown concentrations of cytokines were determined by performing linear regression analysis.

### 2.10 Real-time PCR gene expression

Total RNA was extracted from snap frozen lung tissues and cells using Qiagen RNeasy RNA isolation kit (Qiagen, Germantown, MD) and cDNA was synthesized using High-Capacity cDNA Reverse Transcription Kit (Thermo Fisher Scientific, Waltham, MA). Sequences of PCR primers are provided in Supplementary Information Table 2*.* PCR reaction was performed in triplicate using AriaMX real time PCR machine (Agilent, Santa Clara CA) using SYBR green chemistry, as previously described [36]. Relative expression level of genes of interest was measured using the comparative threshold method with 18S as internal control. Data was analyzed using ΔΔCt method, where fold change = 2^−ΔΔCt^.

### 2.11 Flow cytometry for cellular oxidants

RAW 264.7 cells were treated with CB and CB_ox_ particles and oxidative stress was also analyzed by flow cytometry (using dihydroethidium, DHE), Invivogen, San Diego, CA). Cells that were exposed for 4 h were harvested, centrifuged for 5 min at 400 g and stained with DHE at a concentration of 5 μM in warm Hank's Balanced Salt Solution for 30 min in 37 °C. Cells were washed twice with Phosphate Buffered Saline and an increase in fluorescence was measured using BD-FACS Aria III equipment at 488 nm excitation and 615 nm emission wavelength.

### 2.12 Western blot

Cells were lysed 4 h post particle treatment using RIPA buffer (Thermo Fisher Scientific, Waltham, MA) supplemented with protease and phospatase inhibitor cocktail (Sigma-Aldrich, St. Louis, MO). Total Protein was quantified using BCA (bicinchoninic acid) assay (Thermo Fisher Scientific, Waltham, MA), and proteins were separated electrophoretically in 4–12% bis-tris polyacrylamide gel followed by transfer to PVDF membrane. Membranes were blocked using 3% bovine serum albumin (BSA) and incubated overnight at 4 °C with 1:1000 dilution of primary rabbit monoclonal antibodies (cat# 9251 phospho-JNK, cat# 9252 JNK, cat# 3033 phospho–NF–kβ p65 and cat# 4764S Total- NF-kβ p65, Cell Signaling Technologies, Danvers, MA). Membranes were washed with tris buffered saline -tween solution and conjugated with anti-rabbit HRP conjugated secondary antibody for 1 h (1:10,000) (Cell Signaling Technologies, Danvers, MA). After washing, chemiluminescent signal was developed using ECL prime (Thermo Fisher Scientific, Waltham, MA) and detected using Amersham Imager 600 (Cytiva, Life Sciences, Marlborough, MA) imaging system. β-actin (1:1000) (catalog number sc-47778, Santa Cruz, Dallas, TX) was used as a loading control. The western blots were quantified using ImageJ software (NIH, Bethesda, MA) following the previously described method [55]. Briefly, the images were converted to 8 bit using the ImageJ software. Band density was normalized to the density of the loading control (β-actin) and then plotted as relative density of the phospho to total protein levels.

### 2.13 NF-kβ/AP1 reporter assay

NF-kβ/AP-1 activity was measured using RAW-Blue™ cells (InvivoGen, San Diego, CA). NF-kβ activity causes SEAP production which can be quantified spectrophotometrically by QUANTI-Blue™ (Invivogen, San Diego, CA solution. Briefly, 25,000 cells were seeded in a 96-flat bottomed well plate. After 24 h, cells were treated with CB or and CB_ox_ (50 μg/mL) in DMEM (without phenol red) (Sigma-Aldrich, St. Louis, MO) for 16 h. Supernatant was collected and SEAP levels were measured spectrophotometrically at 655 nm wavelength using SpectraMax®iD5 (Molecular Devices, CA).

### 2.14 *In vitro* permeability assay

FITC-dextran based permeability assay was performed using In vitro Vascular Permeability Assay kit (EMD Millipore, Burlington, MA) as per the manufacturer's instructions. Briefly, C166 cells were grown to monolayer on collagen-coated cell culture inserts for 72 h. Monolayer formation was verified using microscopy. Cells were exposed to conditioned medium for 24 h, after which fluorescein isothiocyanate (FITC)-dextran (dilution 1:50) was added and incubated for 40 min to allow the tracer to permeate across the cell layer. The media was collected from the bottom wells, and fluorescence intensities were measured at an excitation wavelength of 485 nm and emission wavelength of 530 nm using SpectraMax®iD5 (Molecular Devices, CA).

### 2.15 Scratch assay

Wound/scratch assay was performed with C166 endothelial cells using silicon culture inserts (Ibidi®, Planegg, Germany). Briefly, cells were grown on inserts for 72 h. The polymer separation was removed, and cells were treated with conditioned medium as described above. Images were captured after time 0 and 24 h with a 10X objective using Zeiss Tissue Culture Scope (Zeiss Microscopy, Germany). Changes in migration area were quantified using ImageJ software (NIH, Bethesda, MA).

### 2.16 Statistical analyses

Data are presented as mean ± standard error of mean (SEM) from at least three experiments with three technical triplicates in each group. Statistical differences were inferred using analysis of variance (one-way or two-way depending on the experimental design) and Tuckey's post-hoc test. The individual groups were compared by Student-t test. Differences between the groups were considered statistically significant if the p-value was less than or equal to 0.05 (95% confidence level). Statistics were performed using GraphPad Prism v8.3 (GraphPad Software, San Diego, CA).

## 3 Results

### 3.1 CB particles characteristics

CB nanoparticle physicochemical characteristics have been previously reported by our group and others [32,33,[35], [36], [37]]. Briefly, primary particles are round/irregular in morphology and form loosely bound agglomerates. Transmission electron microscopy analyses revealed primary particles size to be 14 ± 6 nm. Specific surface area measurement of CB by Brunauer, Emmett and Teller (BET) method was 274 ± 27 m^2^/g. Particles demonstrated no extractable polycyclic aromatic hydrocarbon (PAH) contents after 8 h of toluene extraction (manufacturer data). Mass spectrometry analysis indicated no detectable metal impurities in the particles. Endotoxin levels were 0.15 EU/mg as demonstrated by the Limulus Amebocyte Lysate (LAL) assay.

### 3.2 Colloidal characteristics of CB and ozone reacted CB particles

**Fig. 1:**
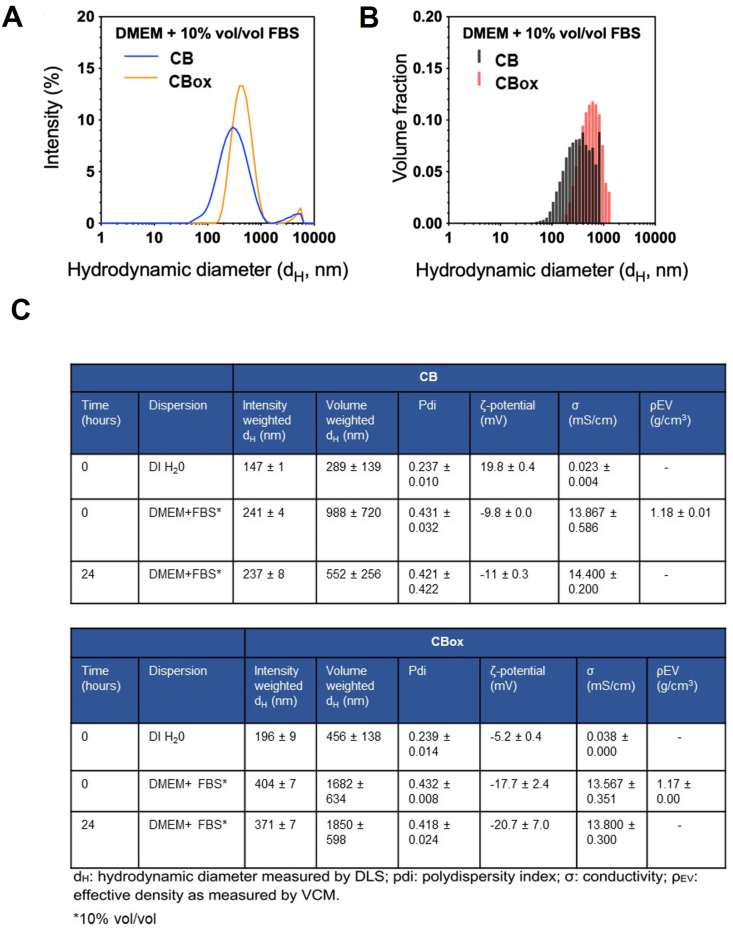
**Colloidal characteristics of CB and ozone reacted CB(CB**_**OX**_**)**. A) Intensity-weighted hydrodynamic diameter distributions of CB and CB_OX_ dispersions in DMEM +10% vol/vol FBS. B). Volume-weighted hydrodynamic diameter distributions of CB and CB_OX_ dispersions in DMEM +10% vol/vol FBS. C) tabulated data on colloidal characteristics of CB and CB_OX_.

The DSE_cr_ for 0.5 mg/mL aqueous dispersion of the CB and CB_OX_ samples were calculated to be 151 and 340 J/mL, respectively. The effective densities of the CB and CB_OX_ particle samples as measured by the Harvard VCM were 1.18 ± 0.01 and 1.17 ± 0.00 g/cm^3^, respectively [44]. Fig. 1A and B respectively present the intensity-weighted and volume-weighted hydrodynamic size distributions of the particle suspensions in DMEM + FBS 10% (vol/vol) as measured by dynamic light scattering (DLS) at 0 h. The hydrodynamic size and stability of CB and CB_OX_ suspensions in water and in DMEM + FBS 10% (vol/vol) as measured by DLS at time 0 and at 24 h is presented in a tabulated form as Fig. 1**C.** Interaction with O_3_ significantly increased the negative charge on the particle surface (zeta potential) in suspension especially in water (19.8 ± 0.4 to −5.2 ± 0.4) causing a net near neutral charge and increased agglomeration. Moreover, CB particles formed smaller aggregates/agglomerates in suspension compared with CB_ox_ particles.

### 3.3 Partico-kinetics and *in vitro* cellualar modeling

**Fig. 2:**
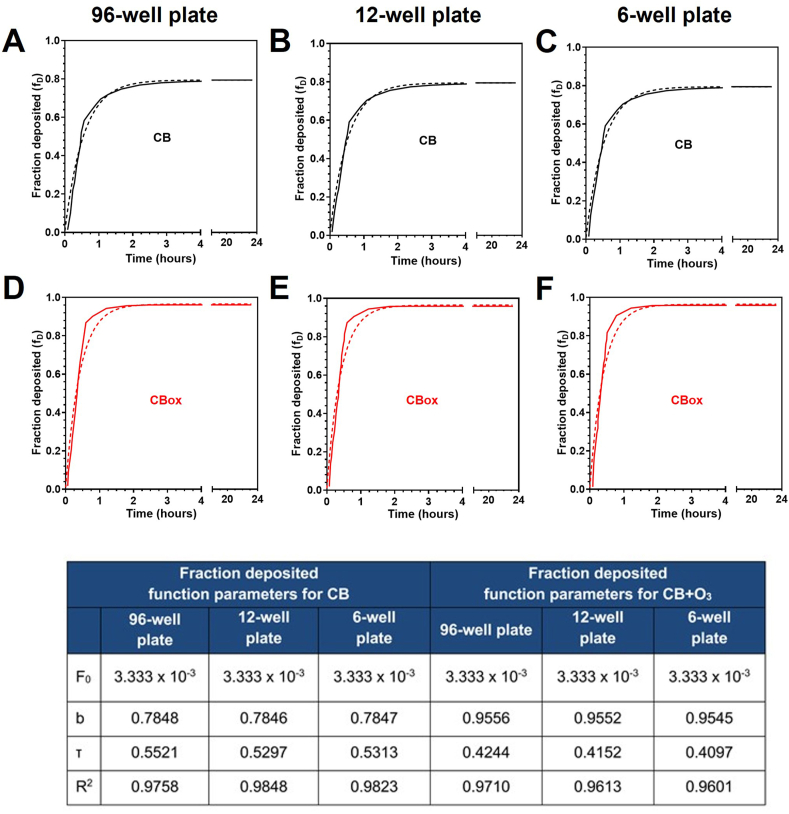
**Fate and transport modeling.** Deposited mass fraction at the cell-particle interface over time for CB (A–C) and CB_OX_ (D–F) for 96-, 12-, or 6-well plates. Continuous lines correspond to the DG model predictions based on experimental data; dashed lines trace the regression modeling.

The effective densities and volume weighted size distributions were used to determine CB and CB_OX_ delivered to cell dose as a function of exposure time using the DG model. Fig. 2 presents the automatically generated deposited mass fraction vs. time for both types of particles, as calculated by the DG model for 6, 12, and 96-well plates. It appears that the sedimentation of agglomerate does not reach an equilibrium within 24 h. For all types of well plates, the mean particle fraction deposited for CB and CB_OX_ over 24 h tends asymptotically to 0.80 and 0.96, respectively (Supplemental Table 1).

### 3.4 Surface functional group alterations on CB_ox_ NPs

Fourier-transform infrared spectroscopy (FTIR) was performed on the CB and CB_ox_ particle samples to characterize the change in chemical functionality. Supplementary Fig. S1 shows the spectra between 800 and 1800 cm^−1^. The spectra of CB_ox_ significantly differs from CB. Functional groups qualitatively identified include C=C, C–O, C=C stretching and C=O. The appearance of a strong absorption at 1660–1770 cm^−1^ in ozone treated CB is a clear indication of enhanced presence of carbonyl group. XPS analyses revealed 5.7% increased O:C ratio. Deconvolution of O1s and C1s peaks from CB and CB_ox_ samples by XPS further confirmed the appearance of surface functional moieties (Supplementary Fig. S1).

### 3.5 Oxidant generation on CB_ox_ NPs

**Fig. 3:**
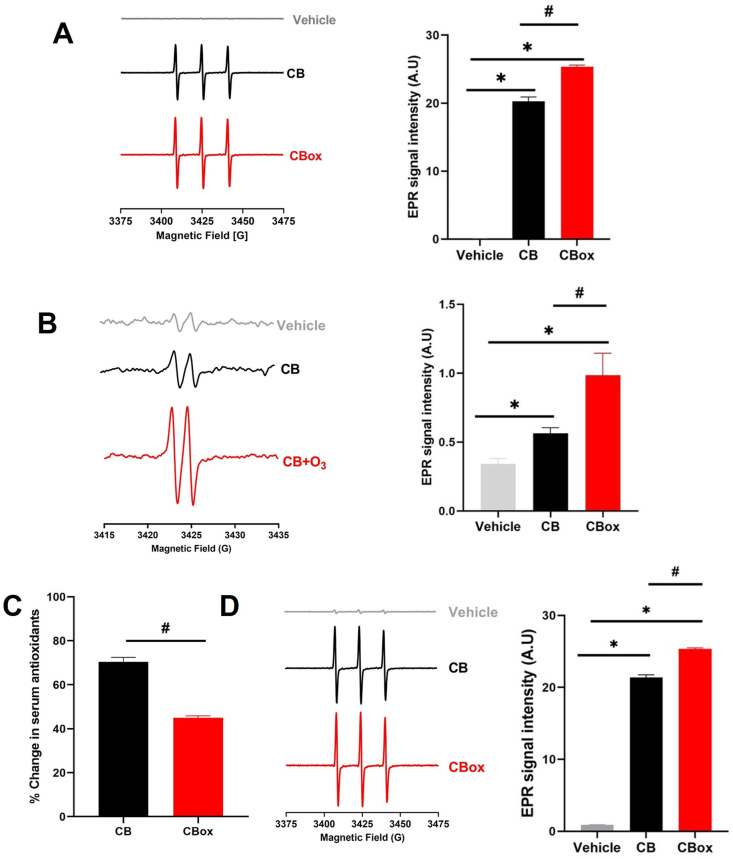
**Increased oxidative potential CB**_**ox**_**NPs compared with CB.** A) Representative room temperature X-band EPR spectra of CM•. EPR spectra of Control, CMH without particles in PBS, CB and ozone interacted CB (CB_OX_) suspensions (50 μg/mL in PBS pH 7.6). The signal intensity was generated using first peak (low field) height of the EPR spectrum. Histogram present quantification of signals. B) Representative room temperature X-band EPR spectra of ascorbate radical production. EPR spectra of Control, CMH without particles in PBS, CB and ozone interacted CB (CB_OX_) suspensions (50 μg/mL in PBS). The signal intensity was generated using first peak (low field) height of the EPR spectrum. Histogram present quantification of signals. C) Ferrous reducing ability of serum (FRAS) assay of CB and CB_OX_ powders. D) Representative room temperature X-band EPR spectra of CM•. EPR spectra of Control, CMH without particles in serum, CB and ozone interacted CB (CB_OX_) suspensions (50 μg/mL in serum). The signal intensity was generated using first peak (low field) height of the EPR spectrum. Histogram represents quantification of signals. Data are presented as mean ± standard error of mean of three independent experiments. Data analyzed by One-way ANOVA followed by Tukey's post-hoc test. *p < 0.05 vs control and #p < 0.05 between CB and CB_OX_.

The oxidant potential of the ozone reacted CB particles was further studied using electron paramagnetic resonance (EPR) spectroscopy (Fig. 3A–B). We first evaluated the reactive surfaces of the CB particles using spin probe CMH and identified significantly greater reactive surfaces with CB_ox_ particles compared to CB. The EPR spectra of CB and ozone-interacted CB incubated with CMH in PBS for 30 min at 37 °C are shown in Fig. 3A. The EPR spectrum exhibits a characteristic triplet pattern with an isotropic hyperfine coupling constant of 16 G [56]. In addition, a significantly greater ascorbate radical EPR signal from the ozone interacted CB particles further confirmed increased oxidant generation potential compared with CB particles (Fig. 3B). *Ferric Reducing Ability of Serum (FRAS)* assay indicated increased potency of CB_ox_ particles to reduce antioxidants in the human serum (Fig. 3C). To further validate these findings and to confirm whether the reduction of antioxidants happened in parallel to/as a result of oxidant generation, we performed EPR analyses on human serum exposed to particles. In concurrence with FRAS, our data clearly indicated a significantly greater oxidant generation in human serum after incubation with ozone interacted CB particles (Fig. 3D).

### 3.6 Increased in vivo biological potency of CB_ox_ particles

**Fig. 4:**
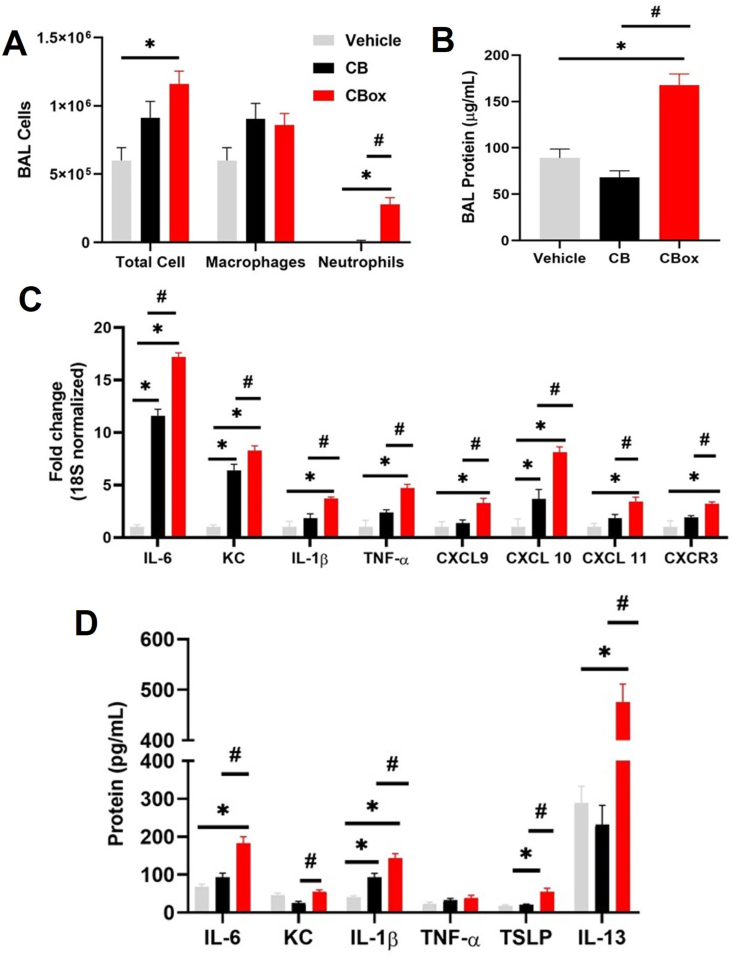
**Ozone reacted carbon black (CB**_**OX**_**) particles induce greater inflammatory response *in vivo* compared to CB.** C57Bl/6J mice were oropharyngeally exposed to 20 μg of particles in (in 50 μl of saline) and euthanized 24 h later. A) Bronchoalveolar lavage (BAL) total cells, macrophages and neutrophils B) total protein contents in BAL fluid C) Real time RT-PCR mRNA expression of IL-6, KC, IL-1β, TNF-α, CXCL9, CXCL10, CXCL11 and CXCR3 in lung homogenates D) Inflammatory cytokine quantification in BAL fluid by ELISA. Data are presented as mean ± standard error of mean with N = 5–6 mice per group. Data analyzed by ANOVA followed by Tukey's post-hoc test. *p < 0.05 vs control and #p < 0.05 between CB and CB_OX_.

A significantly greater number of bronchoalveolar lavage (BAL) neutrophils were observed in mice exposed to CB_ox_ particles compared to CB particles (Fig. 4 A). A significant increase in BALF protein content indicated an increased permeability of air-blood barrier in the lungs (Fig. 4 B). Real-time PCR analysis of lung homogenate further confirmed that CB_ox_ particles induced a greater pro-inflammatory gene expression compared to CB particles (Fig. 4C). A significant elevation in mRNA expression of IL-6, CXCL9, CXCL10, CXCL11, IL-1β, and TNF-α was detected in mice exposed to CB_ox_ particles compared to CB particles. ELISA analysis of various BALF cytokines showed a significant increase in protein concentration of IL-6, IL-1β, TNF-α and KC, indicating a higher inflammatory response in mice exposed to CB_ox_ particles compared to CB (Fig. 4 D).

### 3.7 CB_ox_ particles induce greater inflammatory response and oxidative stress

**Fig. 5:**
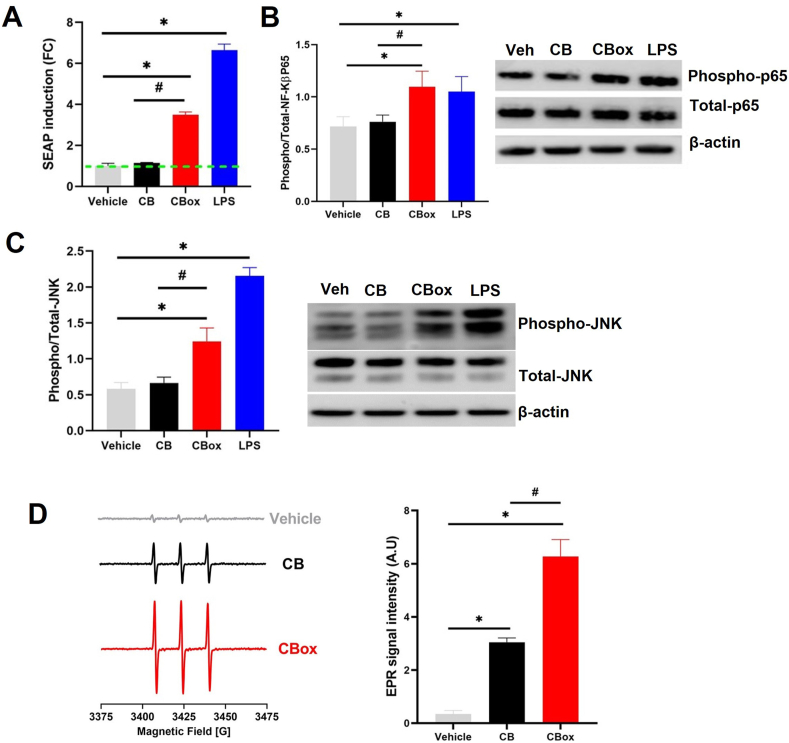
**CB**_**ox**_**particles induces higher inflammation in murine macrophages (RAW 264.7cells) compared to CB particles** A) SEAP reporter assay for NF-kβ activity assay utilizing RAW Blue™ cells. RAW Blue™ cells were exposed to or CB_ox_ particles (50 μg/mL) for 4 h before collecting and supernatants were collected for SEAP activity assay (NF-kβ activity). B) Phosphorylation of p-65, C) Phosphorylation of JNK, D) Representative EPR spectra of vehicle/control (RAW 264.7 cells incubated with 0.2 mM CMH), CB (CB treated with RAW 264.7 cells and incubated with 0.2 mM CMH), CBox (ozone exposed CB treated RAW 264.7 cells incubated with 0.2 mM CMH). The signal intensity was generated using first peak (low field) height of the EPR spectrum. Histogram present quantification of EPR signals. For these EPR study, RAW 264.7 cells were incubated with CMH probe for 30 min at 37 °C and collected for experiments. RAW 264.7 cells were exposed to (vehicle or 50 μg/mL particles) for 4 h and cell lysates were collected for Western blot analyses for phospho/total p65 subunit of NF-kβ and phospho/total JNK. Data are presented as mean ± standard error of the mean of three independent experiments each with triplicate of each condition. Data analyzed by ANOVA followed by Tukey's post-hoc test. *p < 0.05 vs control and #p < 0.05 between CB and CB_OX_.

We utilized mouse macrophage cell line (RAW 264.7) to elucidate signaling pathways activated by the particle exposure. Further RAW-Blue™ (NF-kβ SEAP Reporter cell line) was used to quantify NF-kβ/AP1 activity as SEAP production occurring under the control of NF-kβ and AP-1. A significantly greater SEAP indicated increased activation of NF-kβ/AP-1 by ozone reacted CB particles compared to CB particles and vehicle (Fig. 5A). These results were further validated by Western blot analysis confirming increased phosphorylation of p65 subunit of NF-kβ (Fig. 5B). JNK, a protein involved in oxidant response through mitogen activated protein kinase (MAPK) pathway, was significantly phosphorylated after treatment with CB_ox_ (Fig. 5C). EPR spectroscopy using spin probe CMH on RAW 264.7 cells exposed to the CB_ox_ particles confirmed the significant cellular oxidants generated by CB_ox_ *in vitro*. (Fig. 5D).

### 3.8 Inhibition of oxidant production and JNK protects cells from oxidative stress and inflammation induced by CB_ox_

**Fig. 6:**
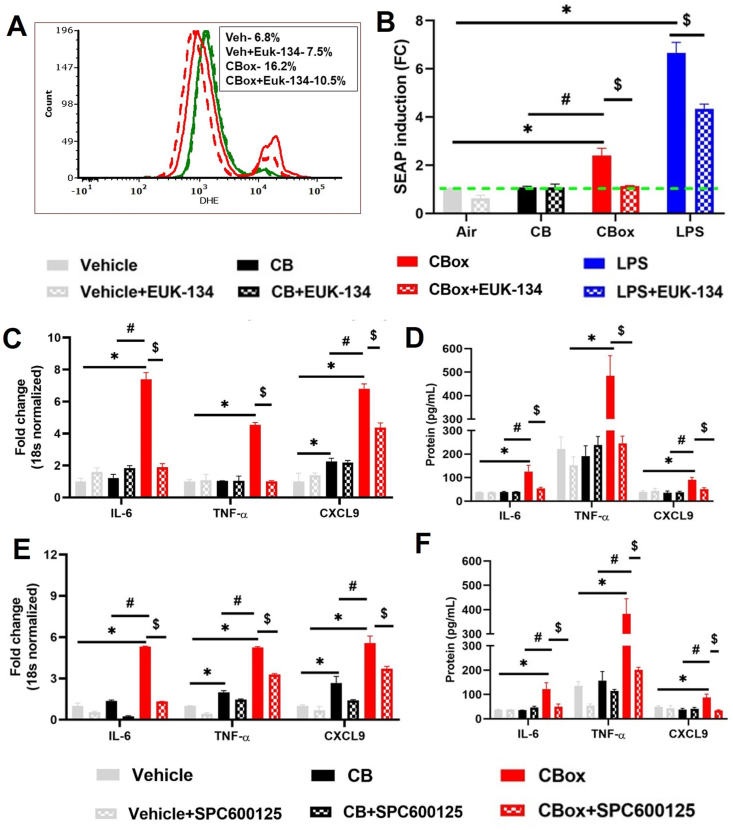
**EUK-134 (catalase-superoxide dismutase mimetic) and** SPC600125 **(JNK inhibitor protects from inflammatory impacts of ozone reacted CB particles.** Pre-treatment with EUK-134 (5 μM) 30 min before particle exposure protects from increased A) Intracellular oxidant generation (dihydroethidium labeling) by flow cytometry, B) SEAP secretion (NF-kβ activity), C) Pro-inflammatory mRNA expression and D) cytokine secretion in cell culture supernatants. Pre-treatment with SPC600125 (20 μM) 30 min before particle exposure protects from increase in E) Pro-inflammatory mRNA expression by real time RT-PCR and F) cytokine secretion by ELISA in cell culture supernatants. After 30 min of pre-treatment cells were exposed to CB or ozone reacted CB particles (50 μg/mL) and supernatant/cells collected after either 4 h (for flow cytometry, SEAP reporter assay and mRNA expression) or 24 h (ELISA assay). LPS (10 ng/mL) was used as positive controls. Data are presented as mean ± standard error of the mean of three independent experiments with triplicates of each condition. Data analyzed by two-way ANOVA followed by Tukey's post-hoc test. *p < 0.05 vs control, #p < 0.05 between CB and CB_OX_ and, $ p < 0.05 between without and with pharmacological agents (EUK-134 and SPC600125).

EUK-134, a synthetic catalase-superoxide dismutase mimetic was utilized to elaborate the role of ozone treated CB particles in oxidant generation. Pre-treatment of RAW 264.7 cells with EUK-134 robustly protected the cells from CBox particle-mediated toxicity. DHE analyses demonstrated EUK-134 treatment significantly protected the cells from CB_ox_ particle-mediated oxidant stress (Fig. 6A, Supplementary Fig. S2). Further, treatment with EUK-134 resulted in significant decrease in NF-kB/AP-1 activity (as evident by reduced SEAP production) (Fig. 6B). LPS was used as a positive control for NF-kB activation. Apart from decrease in oxidative stress and NF-kB/AP-1 activation, EUK-134 treatment also significantly decreased in mRNA expression of inflammatory mediators IL-6, TNF-α, CXCL9, CXCL10, and CXCL11) (Fig. 6C, Supplementary Fig. S3). This was confirmed by decrease in protein level of TNF-α, CXCL9 and IL-6 in cell culture supernatants pre-treated with EUK-134 (Fig. 6D). SPC600125, a potent JNK inhibitor, was utilized to understand the role of JNK signaling pathway in CBox particles induced inflammation. We observed a significant decrease in mRNA expression of IL-6, TNF-α, CXCL9, CXCL10 and CXCL11 after treatment with SPC600125 (Fig. 6E, Supplementary Fig. S5). A significant decrease in TNF-α, CXCL9 and IL-6 protein levels was measured in the cell culture supernatants after 24 h of treatment, establishing the role of JNK in CB_ox_ particles-induced inflammation (Fig. 6F).

**Fig. 7:**
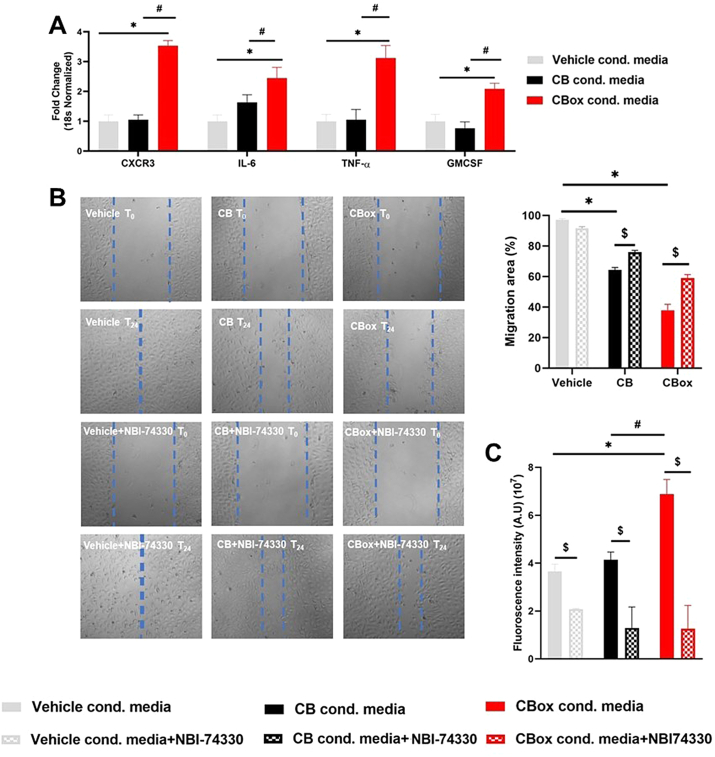
**Endothelial CXCR3 inhibition improves particle-induced impairment of endothelial cell dysfunction.** Conditioned media was collected from RAW 264.7 cells treated for 24 h with vehicle, CB or CB_ox_ particles (50 μg/mL). After testing for no adverse outcome, endothelial cells were exposed to four times diluted conditioned media with or without NBI-74330 (100 nM). A) Pro-inflammatory mRNA expression B) Scratch assay was performed using ibidi wound healing μ plates and images were captured (10X magnification) at 0 h and 24-h post scratch. Assay was repeated 2 times with triplicate of each condition each time. % wound closure was calculated using Image J software. C) FITC-dextran based permeability assay. Data are presented as mean ± standard error of mean. Data analyzed by two-way ANOVA followed by Tukey's post-hoc test. *p < 0.05 vs control, #p < 0.05 between CB and CB_OX_ and, $ p < 0.05 between without and with NBI-74330.

**Macrophage-secreted soluble mediators impact endothelial cell responses through CXCR3 pathway.** In order to understand the influence of macrophage-secreted factors on endothelial cell responses, RAW 264.7 cells were challenged with CB_ox_ particles for 24 h and supernatants (conditioned media containing the secreted factors) were collected. This media was diluted 1:4 with fresh media and was used to challenge endothelial cells. CB_ox_ particle conditioned medium from RAW 264.7 cells significantly upregulated inflammatory gene mRNA expression (IL-6, TNF-α, and GM-CSF) in endothelial cells (Fig. 7A). We also observed a significant upregulation of CXCR3 mRNA. Compared to the vehicle control, both CB and CBox particle treated cell conditioned media impaired the endothelial wound healing (measured by scratch assay) (Fig. 7B). CB_ox_ induced significantly greater scratch healing impairment. This impairment was significantly improved by addition of CXCR3 antagonist (NBI-74330). Challenge with the conditioned media significantly increased endothelial monolayer permeability that was significantly reduced by addition of NBI-74330 (Fig. 7C) confirming the role of macrophage secreted factors in inducing endothelial monolayer permeability.

## 4 Discussion

Herein, we demonstrate that CB particles, after interaction with O_3_, have altered surface chemistry characteristics and demonstrate an increased capacity for oxidant generation. Interaction with O_3_ resulted in not only changes in surface oxidation but also the colloidal characteristics which also affect their delivered to cell dose over the 24 h exposure period. Compared to CB, CB_ox_ particles displayed a negative charge/zeta potential due to development of negatively charged functionalities. These functionalities balance the positive charge in the starting material and thus result in a more neutral charge leading to an increased hydrodynamic diameter of CB_ox_ particles. These findings are in agreement with previously reported observations [10]. Fate and transport *in vitro* particle dosimetric modeling indicated a slightly greater deposition fraction for CB_ox_ particles compared to CB. We performed equivalent deposited dose experiment to determine if this change in deposited dose has an influence on the outcomes (Supplementary Fig. S5). Our results robustly reflect that even at the similar deposited to cell dose, CB particles did not induce increases in mRNA expression of inflammatory genes compared to CB_ox_ particles. FTIR analyses demonstrated that interaction with O_3_ leads to altered surface functional group composition. The appearance of absorptions at 1630 and 1730 cm^−1^ in CB_ox_ is an indication of enhanced presence of carbonyl and conjugated carbonyl groups. Absorption at 1240 cm^−1^ potentially evolves from lactones, ethers, and the symmetric bend of hydrogen atoms on adjacent double-bonded carbon atoms [10,57]. O_3_ treatment-mediated oxidation of the CB particles induced the appearance of new bands between 1420 and 1580 cm^−1^. Oxidation-dependent appearance of these new bands has also been previously observed with CB [58,59]. Similarly, XPS analyses of O1s peak indicated high intensity bands on CBox surface at ∼531, 532 and 533 eV which corresponded to C–O, -C-C=O, C=O, and C–O (from C–O–C and C–OH) functional groups [3,60,61]. Deconvolution of C1s peak demonstrated peaks at ∼284 eV, 285, and, 288 eV corresponding to C–C/C–H bonded carbon, C–O, and -O-C=O (carbonyl carbon)/C=O/carboxyl carbon, respectively [3,10,60,61].

As demonstrated by the FTIR analyses, there are significant increases in functional groups (carbonyls, carboxyl, hydroxyl) on the CB_ox_ particles (Supplementary Fig. S1). The appearance of such functionalities and known disorders in the structure of CB particles potentially lead to the formation of free radicals through Criegee intermediate formation [9,10,60]. It is plausible that Criegee intermediates (carbonyl oxides), which were originally described to be produced by interaction of O_3_ with hydrocarbons, are involved in the production of surface radicals [62,63]. Indeed, ozone reaction on the carbon surface by addition across unsaturated carbon bonds can lead to the formation of ozonide, resulting in free radical yield [63]. The graphitic and paramagnetic centers in the carbon are potential sites for such reactions [60]. Interaction of ozone with particles in the air was shown to create molecular oxygen and free radicals (some of them long lived) [64]. Here, using CMH probe and ascorbate, we demonstrated that the ozone treatment increases the reactive species/radicals on the surface of the CB particles (Fig. 3).

This study has implications beyond the oxidized nanoparticle hazard identification. To some extent, similar reactions may potentially occur in the air when carbon-based particulates interact with O_3_ resulting in altered physicochemical characteristics of the particulates [65]. Particulate air pollution also contains significant amounts of aromatic hydrocarbons which are known to form ozonoids after interacting with O_3_ and thus potentially form oxidants through Carigee intermediate-involved chemistry. Moreover, combined exposure to O_3_ and particulate matter/diesel exhaust particles is known to have increased lung/cardiovascular toxicity [65]. We recently demonstrated a significant increase in the biological potency of inhalation co-exposure to CB and O_3_ and occurrence of the surface reactions at doses as low as 200 ppb O_3_ and 250 μg/m^3^ CB [41]. In a similar vein, titanium dioxide (E171) particles were found to compromise the tight junction of intestinal epithelial barrier when co-exposed with pesticides [66,67]. Present findings provide strong evidence that particle surface modifications/oxidation may also be playing an important role in the observed increased biological activity of the co-exposure. The potential synergistic effects from NP and gaseous co-exposures are usually ignored in nanotoxicology research [[68], [69], [70]].

We demonstrated that oxidants (both acellular and cellular), generated by the particles are mediated through NF-kB and JNK signaling in macrophages. This pro-inflammatory signaling was significantly blunted after administration of a synthetic catalase-superoxide dismutase mimetic (EUK134), confirming the role of oxidant production/oxidative stress in the observed pro-inflammatory response. EUK 134 has previously been successfully administered in both *in vitro* and in vivo studies to reduce the oxidant mediated signaling in cardiovascular, urologic and neurologic disorders such as myocardial ischemia reperfusion injury, pulmonary hypertension, ischemic brain injury [[71], [72], [73]]. The role of JNK phosphorylation in inflammatory signaling was confirmed using a specific JNK inhibitor SPC600125. Our results indicate that the inhibition of JNK signaling attenuated inflammatory mRNA (IL-6, TNF-α, GMCSF, CXCL9, TGF-β) and protein (TNF-α, CXCL9, IL-6) expression in RAW 264.7 macrophages. JNK signaling has been previously shown to regulate diesel exhaust particle induced GM-CSF secretion by the airway epithelial cells [74].

Previous work has shown that calcium signaling, mitochondrial alterations and Rho-associated kinase (ROCK) are the mechanistic pathways involved in CB particle exposure related endothelial dysfunction [39,40]. We observed significantly greater CXCL-9 mRNA expression and secretion by macrophages after CB_ox_ particles exposure. We also observed similar increases in CXCL-10 and CXCL-11 mRNA expression in RAW 264.7 cells. These chemokines (CXCL9/10/11) have important roles in chemotaxis of immune cells during the inflammation/wound healing responses and have been shown to inhibit angiogenesis [75,76]. Humans have three isoforms of the CXCR3 receptor which can exert opposing effects depending on the isoform [76,77]. CXCL9/10/11 are known ligands for the CXCR3/GPR9/CD183 receptor [76]. CXCR3 was originally discovered in murine endothelial cells [78]. CXCR3 is a G-protein coupled receptor which is expressed on immune cells as well as epithelial and endothelial cells [79], and is implicated in multitudes of cellular functions including chemotaxis, cellular growth and proliferation, angiogenesis/angiostasis, migration and apoptosis [[80], [81], [82]]. It is interesting to note that some of the pathways influenced by CXRC3 receptor (MAPK, phospholipase 3 and PI3K) are also implicated by ozone-interacted carbon particles [25,26,83]. Endothelial cells migration is an important factor in wound healing and impairment of migration delays wound healing. Endothelial wound healing was significantly delayed by the conditioned medium from CB_ox_ and the response was partially reversed by blocking CXCR3 (using NBI-74330) confirming that macrophage-released soluble factors were impacting migration through CXCR3. Our results are in agreement with previous findings of CXCR3 signaling leading to μ-calpain dependent cell-substrate adhesion molecule cleavage and impairment of endothelial cell migration [84,85]. It has been previously demonstrated that knockout of chemokines or their receptors such as CXCR2 and CXCR3 in a cutaneous model resulted in delayed or incomplete wound healing [86]. Thus, it is probable that the role of CXCR3 and its ligands is cell type specific. Detailed *in vivo* analyses using genetically modified mice for endothelial specific (over expression/knockout) can further clarify the role of CXCR3 and its ligands in endothelial wound healing.

From an environmental perspective, it is important to note that the engineered CB NPs significantly differ from atmospheric black carbon particles (BC) in terms of physicochemical characteristics and chemical composition [87]. While interaction with O_3_ does lead to aging of CB and induces surface functional group alterations, these particles are not considered BC in their entirety as they still demonstrate distinct chemical composition (lack organic components like polycyclic aromatic hydrocarbons, metals, endotoxin) and shape/morphology (homogenous rounded vs irregular). However, considering the known role of particle size in dictating pulmonary toxicity, low toxicity low solubility dust (LTSD) particles are widely used as a surrogate of environmental ultrafine particle exposure. Previous reports on ozone interacted CB (sometimes referred as BC) utilized oxidized particles generated using 100 ppm O_3_ in an N_2_ rich environment) [[25], [26], [27], [28], [29], [30], [31],^88^]. We report here the oxidation of CB by as little as 2 ppm ozone which is routinely reported in numerous of in vivo studies [[89], [90], [91]].

It is known that exposure to CB particles results in systemic cardiovascular effects such as vasomotor dysfunction, endothelial toxicity/activation, and atherosclerosis [38,[92], [93], [94]]. However, the lack of such outcomes on CB_ox_ particles motivated this study. In the current work we evaluated if oxidation further alters the observed CB toxicity and evaluated the mechanism of toxicity. Given the very minute number of ultrafine particles that reach systemic circulation (∼0.3%) after inhalation of the particulate pollutants, there is a greater potential for secreted factors from damaged pulmonary cells indirectly influencing the pulmonary and systemic toxicity. Indeed, the significance of early pulmonary responses in inducing systemic impacts of CB particles has already been demonstrated [95]. Because macrophages are among the first cells to be recruited at particle deposition sites, and are known to be a potent source of cytokine/chemokine production after CB exposure, we evaluated the influence of macrophage secreted factors on endothelial cells [34,96]. Endothelial dysfunction plays a critical role in many cardiovascular pathologies and includes upregulation of adhesion molecules, production of vasoactive molecules and production of inflammatory mediators. RAW 264.7 cells are widely used model for murine macrophages and particle studies. We used C166 cells as a murine endothelial cell model as these retain characteristics of endothelial cells. Moreover, both cell types are cultured in same media and thus present the opportunity to perform mechanistic studies involving secreted factors. Our study is the first to elaborate the impacts of CB_ox_ particles using this conditioned medium approach.

**Fig. 8:**
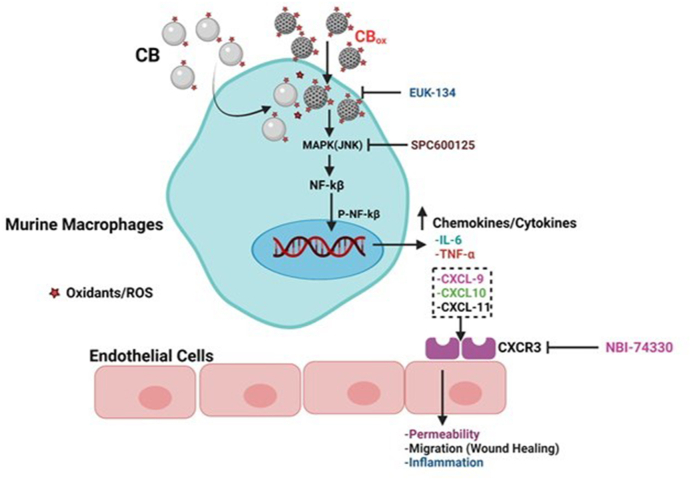
**Overview of macrophage-endothelial cell crosstalk in particle induced toxicity.** Particle induced a cellular and a cellular oxidant generation leads to phosphorylation of JNK and NF-kB resulting in inflammatory cytokine gene and protein expression in RAW 264.7cells. Among the produced chemical mediators CXCR3 agonists (CXCL9, 10 and 11) are prominent. These mediators act on endothelial cells and leads to impaired wound closure and increased monolayer permeability. Endothelial cells CXCR3 inhibition partially prevents these changes and reduces endothelial cell toxicity and pro-inflammatory response.

In conclusion, this study establishes that oxidized CB particles produce acellular and cellular oxidants which are seminal factors for greater *in vitro* and in vivo inflammatory responses compared to CB NPs. We further mechanistically elaborated the role of particle-induced soluble factors (specifically CXCR3 ligands)-released from macrophages in impacting endothelial cell function. The observed pathways impacted by exposure are summarized in the overview figure (Fig. 8). Further mechanistic studies are needed to clarify the pathophysiological consequences of pulmonary CB_ox_ particle exposure in occupational settings and for the general population. Moreover, microvascular functional assessments are needed to elucidate systemic vascular consequences of pulmonary exposure to CB_ox_.

## Disclaimer

The findings and conclusions in this report are those of the author(s) and do not necessarily represent the official position of the National Institutes of Health and National Institute for Occupational Safety and Health, Centers for Disease Control and Prevention. Mention of brand name does not constitute product endorsement.

## CRediT authorship contribution statement

**Nairrita Majumder:** Methodology, Investigation, Formal analysis, Visualization, Writing – original draft. **Murugesan Velayutham:** Methodology, Investigation, Formal analysis, Visualization, Writing – original draft. **Dimitrios Bitounis:** Methodology, Investigation, Formal analysis, Writing – original draft. **Vamsi K. Kodali:** Methodology, Investigation, Formal analysis, Visualization, Writing – original draft. **Md Habibul Hasan Mazumder:** Methodology, Investigation, Formal analysis, Visualization. **Jessica Amedro:** Investigation, Formal analysis, Methodology. **Valery V. Khramtsov:** Methodology, Writing – original draft. **Aaron Erdely:** Methodology, Writing – original draft. **Timothy Nurkiewicz:** Methodology, Writing – original draft. **Philip Demokritou:** Methodology, Investigation, Writing – original draft. **Eric E. Kelley:** Methodology, Investigation, Writing – original draft. **Salik Hussain:** Conceptualization, Methodology, Investigation, Formal analysis, Visualization, Writing – original draft, Supervision, Project administration, Funding acquisition, All authors read and approved the final manuscript.

## Declaration of competing interest

The authors declare that they have no known competing financial interests or personal relationships that could have appeared to influence the work reported in this paper.
