# Supplementary material for: Oxidized carbon black nanoparticles induce endothelial damage through C-X-C chemokine receptor 3-mediated pathway
Source: Redox Biol. 2021 Oct 4;47:102161. doi: 10.1016/j.redox.2021.102161 (PMC8502956; doi:10.1016/j.redox.2021.102161)
Supplement: Multimedia component 1 [file mmc1.pptx]

## Slide 1
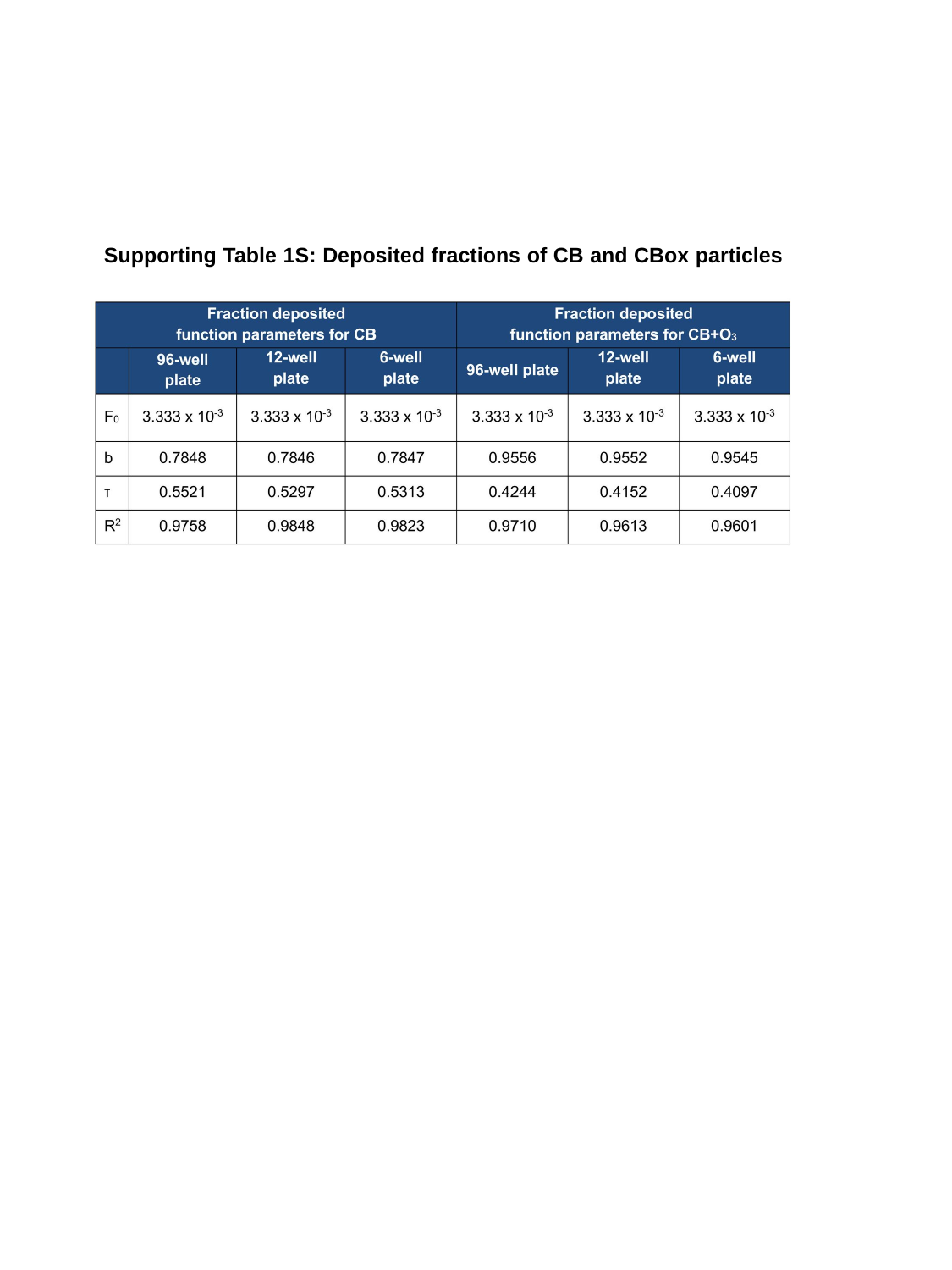

Supporting Table 1S: Deposited fractions of CB and CBox particles

## Slide 2
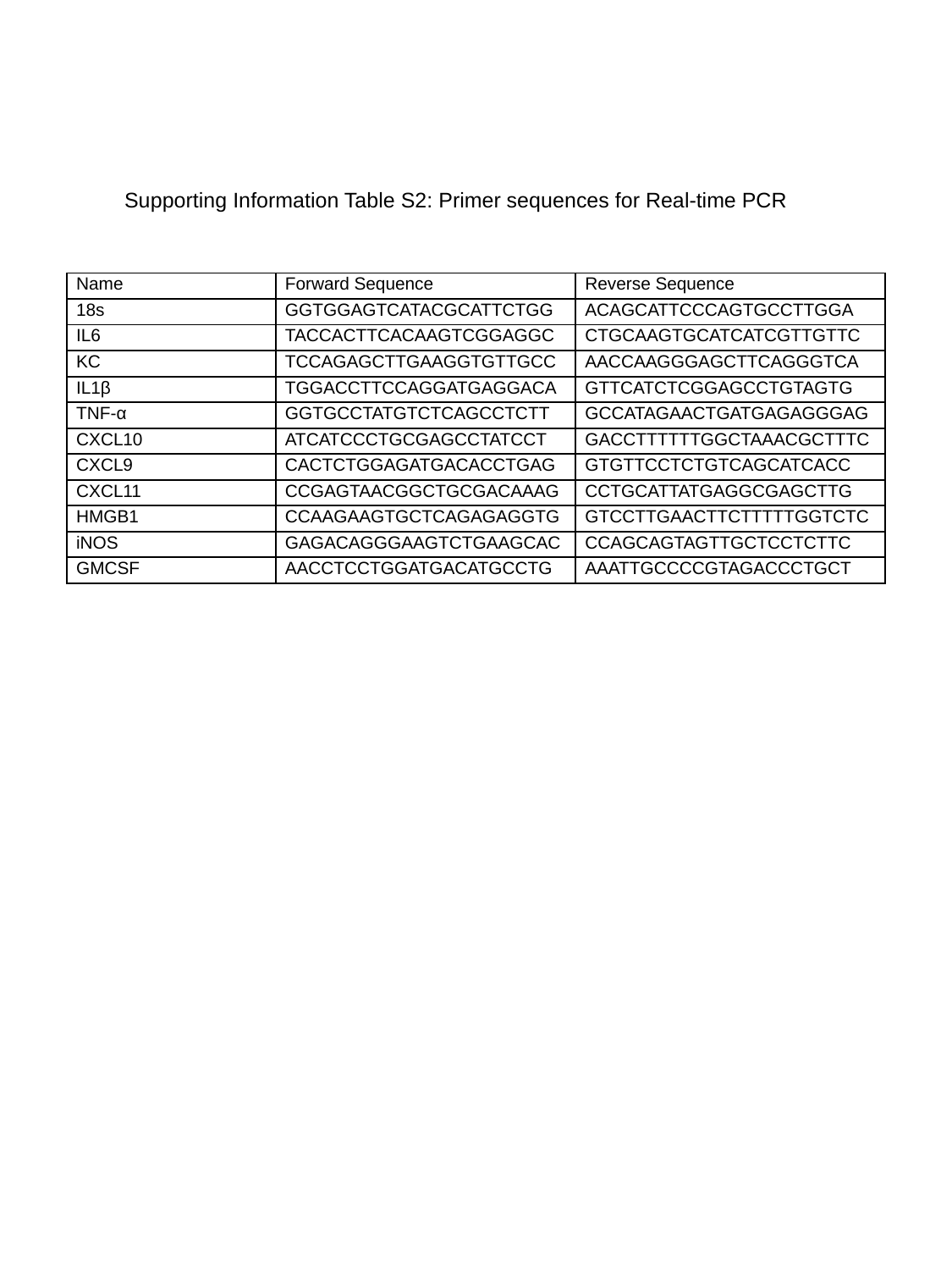

Supporting Information Table S2: Primer sequences for Real-time PCR
| Name | Forward Sequence | Reverse Sequence |
| --- | --- | --- |
| 18s | GGTGGAGTCATACGCATTCTGG | ACAGCATTCCCAGTGCCTTGGA |
| IL6 | TACCACTTCACAAGTCGGAGGC | CTGCAAGTGCATCATCGTTGTTC |
| KC | TCCAGAGCTTGAAGGTGTTGCC | AACCAAGGGAGCTTCAGGGTCA |
| IL1β | TGGACCTTCCAGGATGAGGACA | GTTCATCTCGGAGCCTGTAGTG |
| TNF-α | GGTGCCTATGTCTCAGCCTCTT | GCCATAGAACTGATGAGAGGGAG |
| CXCL10 | ATCATCCCTGCGAGCCTATCCT | GACCTTTTTTGGCTAAACGCTTTC |
| CXCL9 | CACTCTGGAGATGACACCTGAG | GTGTTCCTCTGTCAGCATCACC |
| CXCL11 | CCGAGTAACGGCTGCGACAAAG | CCTGCATTATGAGGCGAGCTTG |
| HMGB1 | CCAAGAAGTGCTCAGAGAGGTG | GTCCTTGAACTTCTTTTTGGTCTC |
| iNOS | GAGACAGGGAAGTCTGAAGCAC | CCAGCAGTAGTTGCTCCTCTTC |
| GMCSF | AACCTCCTGGATGACATGCCTG | AAATTGCCCCGTAGACCCTGCT |

## Slide 3
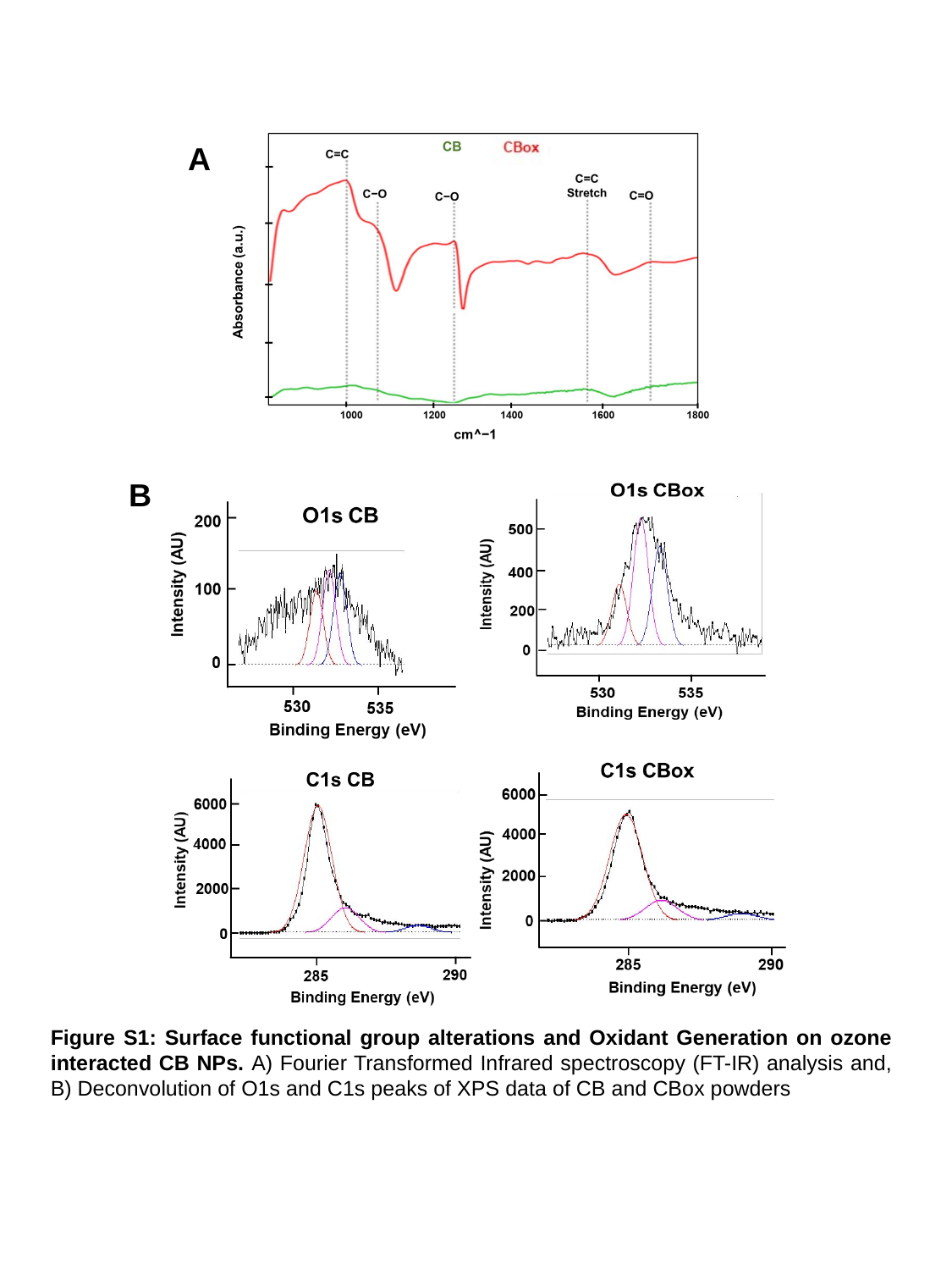

A
B
Figure S1: Surface functional group alterations and Oxidant Generation on ozone interacted CB NPs. A) Fourier Transformed Infrared spectroscopy (FT-IR) analysis and, B) Deconvolution of O1s and C1s peaks of XPS data of CB and CBox powders

## Slide 4
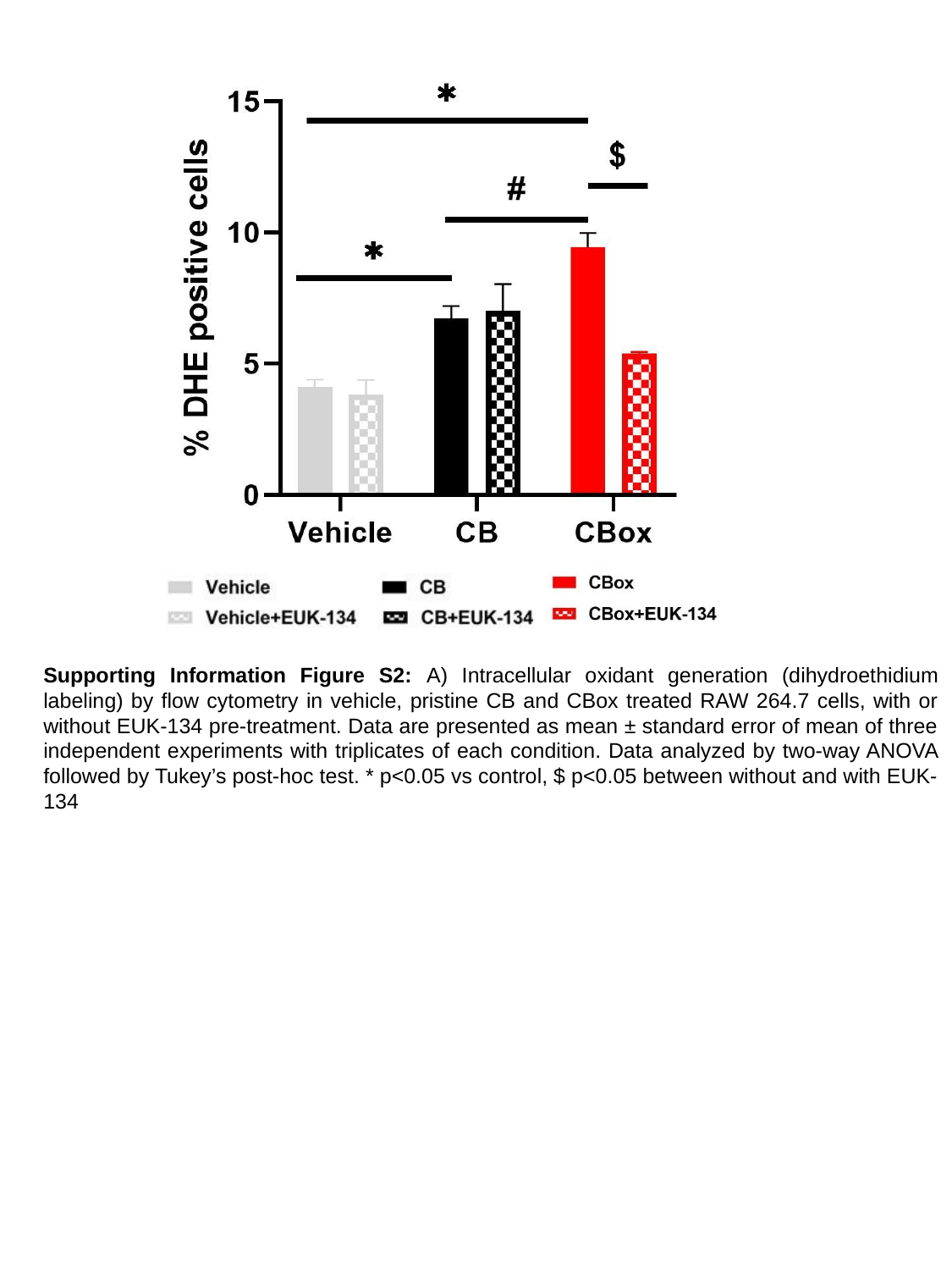

Supporting Information Figure S2: A) Intracellular oxidant generation (dihydroethidium labeling) by flow cytometry in vehicle, pristine CB and CBox treated RAW 264.7 cells, with or without EUK-134 pre-treatment. Data are presented as mean ± standard error of mean of three independent experiments with triplicates of each condition. Data analyzed by two-way ANOVA followed by Tukey’s post-hoc test. * p<0.05 vs control, $ p<0.05 between without and with EUK-134

## Slide 5
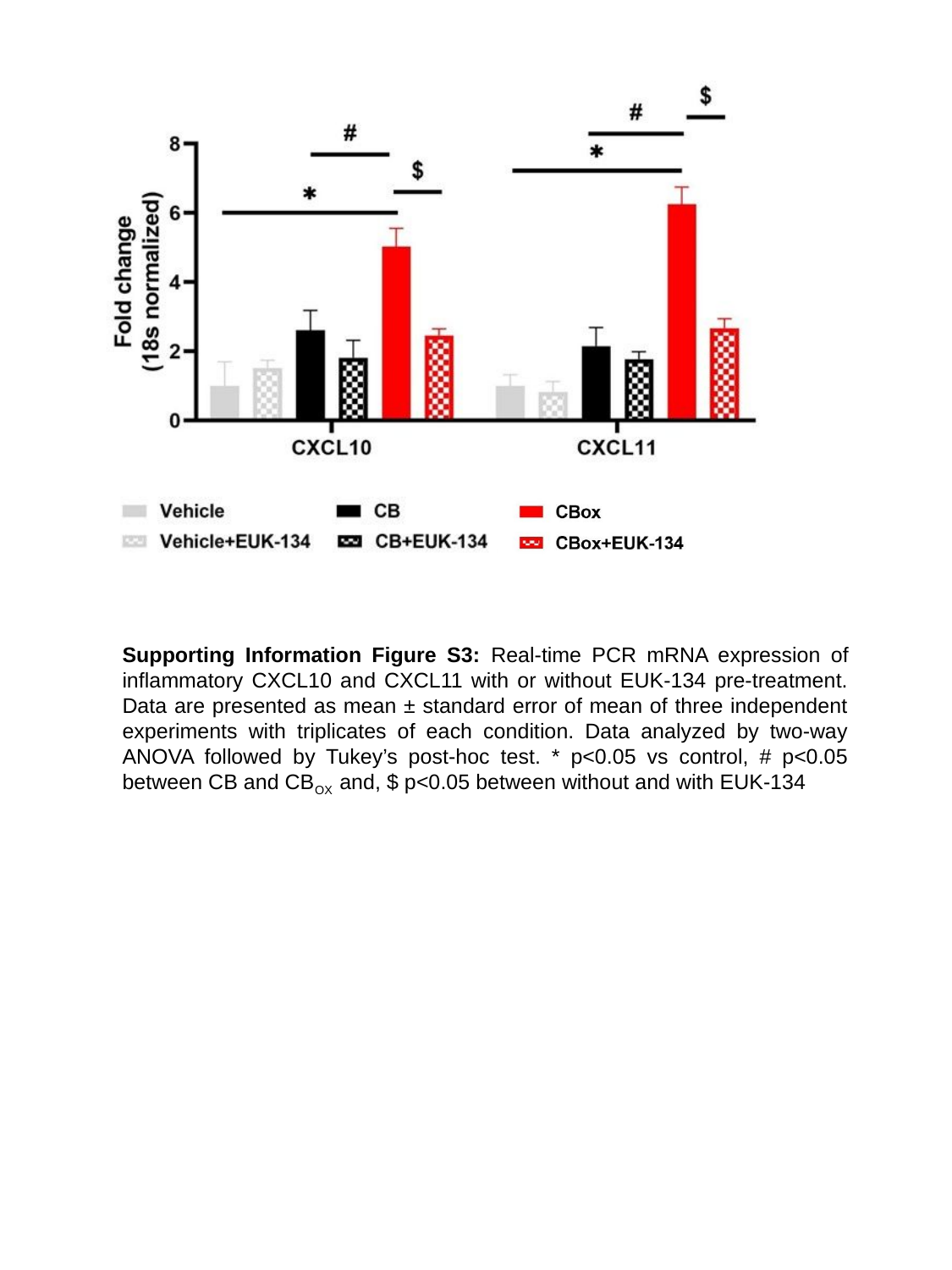

Supporting Information Figure S3: Real-time PCR mRNA expression of inflammatory CXCL10 and CXCL11 with or without EUK-134 pre-treatment. Data are presented as mean ± standard error of mean of three independent experiments with triplicates of each condition. Data analyzed by two-way ANOVA followed by Tukey’s post-hoc test. * p<0.05 vs control, # p<0.05 between CB and CBOX and, $ p<0.05 between without and with EUK-134

## Slide 6
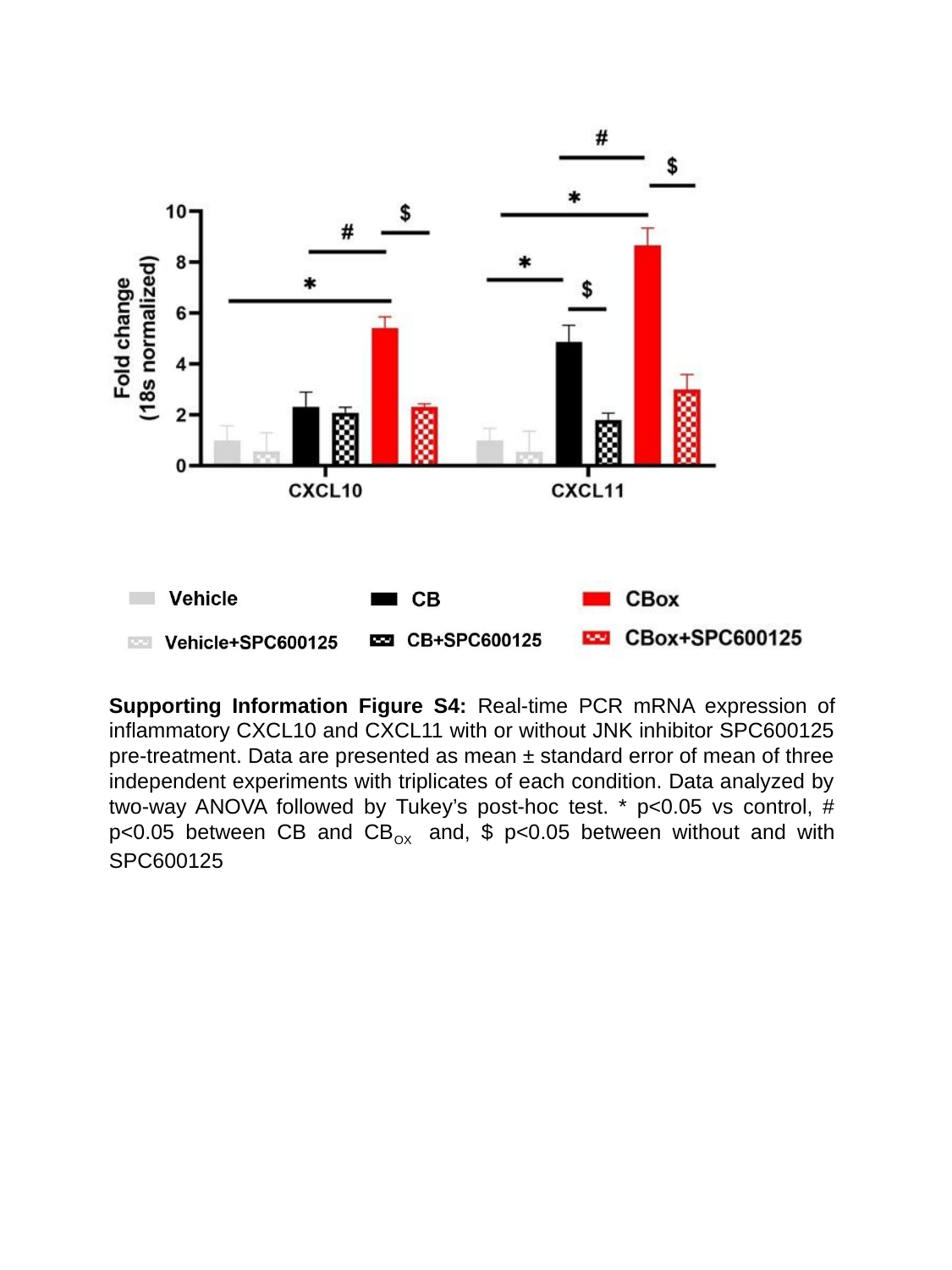

Supporting Information Figure S4: Real-time PCR mRNA expression of inflammatory CXCL10 and CXCL11 with or without JNK inhibitor SPC600125 pre-treatment. Data are presented as mean ± standard error of mean of three independent experiments with triplicates of each condition. Data analyzed by two-way ANOVA followed by Tukey’s post-hoc test. * p<0.05 vs control, # p<0.05 between CB and CBOX and, $ p<0.05 between without and with SPC600125

## Slide 7
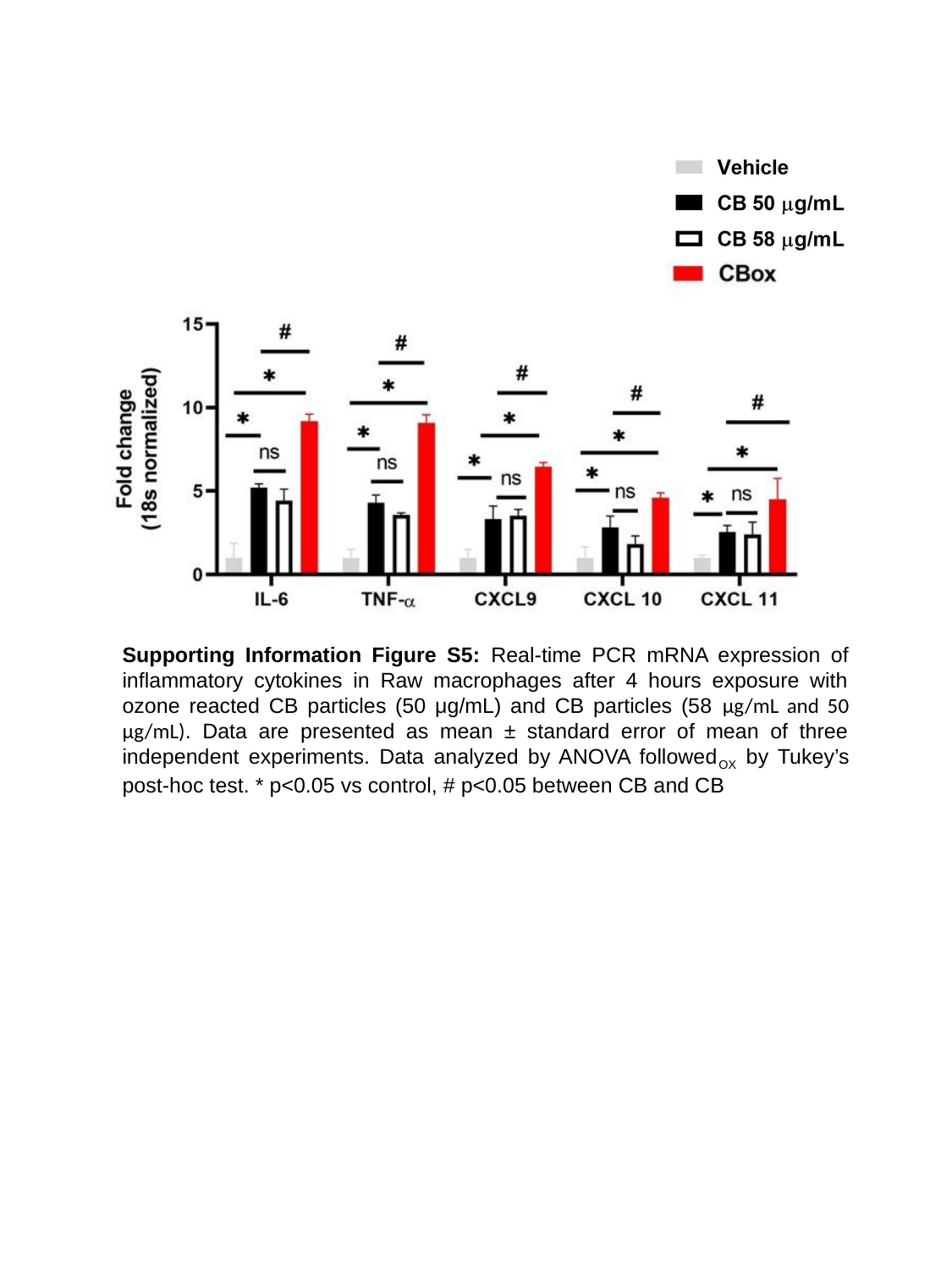

Supporting Information Figure S5: Real-time PCR mRNA expression of inflammatory cytokines in Raw macrophages after 4 hours exposure with ozone reacted CB particles (50 μg/mL) and CB particles (58 µg/mL and 50 µg/mL). Data are presented as mean ± standard error of mean of three independent experiments. Data analyzed by ANOVA followedOX by Tukey’s post-hoc test. * p<0.05 vs control, # p<0.05 between CB and CB
